# Destruction of tumor mass by gadolinium-loaded nanoparticles irradiated with monochromatic X-rays: Implications for the Auger therapy

**DOI:** 10.1038/s41598-019-49978-1

**Published:** 2019-09-30

**Authors:** Kotaro Matsumoto, Hiroyuki Saitoh, Tan Le Hoang Doan, Ayumi Shiro, Keigo Nakai, Aoi Komatsu, Masahiko Tsujimoto, Ryo Yasuda, Tetsuya Kawachi, Toshiki Tajima, Fuyuhiko Tamanoi

**Affiliations:** 1 Institute for Integrated Cell-Material Sciences, Institute for Advanced Study, Kyoto University, Kyoto, Japan; 2 Kansai Photon Science Institute, Quantum Beam Science Research Directorate, National Institutes for Quantum and Radiological Science and Technology, Hyogo, Japan; 3 Center for Innovative Materials and Architectures, Vietnam National University-Ho Chi Minh City, Ho Chi Minh City, Vietnam; 4 Department of Physics and Astronomy, University of California, Irvine, CA USA; 5 Department of Microbiology, Immunology and Molecular Genetics, University of California, Los Angeles, USA

**Keywords:** Radiotherapy, Nanotechnology in cancer

## Abstract

Synchrotron generated monochromatic X-rays can be precisely tuned to the K-shell energy of high Z materials resulting in the release of the Auger electrons. In this work, we have employed this mechanism to destruct tumor spheroids. We first loaded gadolinium onto the surface of mesoporous silica nanoparticles (MSNs) producing gadolinium-loaded MSN (Gd-MSN). When Gd-MSN was added to the tumor spheroids, we observed efficient uptake and uniform distribution of Gd-MSN. Gd-MSN also can be taken up into cancer cells and localize to a site just outside of the cell nucleus. Exposure of the Gd-MSN containing tumor spheroids to monochromatic X-ray beams resulted in almost complete destruction. Importantly, this effect was observed at an energy level of 50.25 keV, but not with 50.0 keV. These results suggest that it is possible to use precisely tuned monochromatic X-rays to destruct tumor mass loaded with high Z materials, while sparing other cells. Our experiments point to the importance of nanoparticles to facilitate loading of gadolinium to tumor spheroids and to localize at a site close to the nucleus. Because the nanoparticles can target to tumor, our study opens up the possibility of developing a new type of radiation therapy for cancer.

## Introduction

Recent advance in Nanotechnology led to the generation of a variety of nanomaterials. Of particular interest to cancer therapy are nanoparticles, nanosize materials with a diameter ranging from 40 to 400 nm^1,2^. Various materials have been used to produce nanoparticles including liposomes, polymers, inorganic materials and proteins. Among inorganic nanoparticles, mesoporous silica nanoparticles (MSNs) are of significant interest for biomedical applications, as they have emerged as a powerful carrier for a variety of compounds^3–5^. MSNs contain thousands of pores that provide a large surface area where a variety of compounds can be absorbed. In fact, the surface area of a gram of MSN has been calculated to encompass more than 500 square meters, providing a vast space for binding various reagents.

Nanoparticles have a number of features that are advantageous for cancer therapy. First, they can be used to deliver materials into cancer cells. For example, many anticancer drugs such as camptothecin are water insoluble and cannot be taken up into cells without the help of nanoparticles. We have shown that MSNs can store camptothecin and efficiently deliver the drug into cancer cells^6,7^. The cellular uptake is due to the use of the endocytosis mechanism which involves endosomal vesicles. This vesicular transport results in delivering nanoparticles to lysosomes which are localized adjacent to cell nucleus. This is where organelles with vital cellular functions are located. In addition, MSNs are efficiently taken up into tumor spheroids as shown in this paper. In animal settings, nanoparticles can accumulate in tumor primarily due to leaky vasculature (enhanced permeability and retention effect) as well as by tumor targeting, suggesting that MSNs can be used to deposit various materials to the tumor^8^.

Monochromatic X-rays may greatly expand the horizon of radiation cancer therapy. Conventional X-rays have broad energy spectra with photons in various energy levels so that when irradiated on the patient the shallower tissues are damaged before they reach to the target tumor. In contrast, the monochromatic X-ray beam can be precisely tuned to target K-shell of high Z atoms (such as gold, silver, iodine, gadolinium and platinum) with specifically large cross-section at that energy of X-rays. The exposure causes ejection of inner K shell electrons (K-edge activation). The resulting electron vacancy needs to be filled with higher shell electrons releasing energy which then causes release of additional electrons from higher shells. Thus, this series of events releases Auger electrons^9–12^. In cancer therapy, this approach has been pursued as photon activation therapy (PAT)^10,11^. DNA damage, cell killing and even tumor inhibition has been studied using compounds such as iodine, platinum and others. However, results on the studies of the Auger effect in biological settings have not been convincing so far. DNA and cell studies showed that the exposure to monochromatic X-rays causes enhancement of DNA breaks^13–17^, but, the extent of enhancement was rather modest^10^. Rat glioma models were used to examine effect of platinum compounds (cisplatin and carboplatin) combined with synchrotron X-rays on tumor growth^18–21^, however, this effect may be largely due to DNA adduct formation^20^.

In this work, we set out to test the idea whether the use of nanoparticles can improve the photon activation therapy. In particular, we speculated that the use of nanoparticles will provide a situation where high Z atoms are located close to the cell nucleus in a concentrated fashion inside cancer cells. For the high Z material, we used gadolinium that has been studied for their ability to generate Auger electrons and to cause DNA damage^22,23^. In addition, gadolinium is used as MRI enhancing agent^24^ raising the possibility that cancer therapy can be coupled with diagnosis. Monochromatic X-rays of defined energy were obtained by SPring-8 synchrotron facility and a set up was designed for this experiment. As for the source of tumor sample, we used tumor spheroid^25^, a convenient and versatile model for human tumor, that is formed by carrying out three-dimensional culture of human cancer cells.

We report that gadolinium loaded mesoporous silica nanoparticles are efficiently taken up into cancer cells and tumor spheroids. Irradiation with monochromatic X-rays results in complete destruction of the tumor spheroids. Importantly, this effect required a precise energy level that corresponds to the level just above the K-edge energy of gadolinium.

## Results

### X-ray irradiation experimental setup and gadolinium characterization

**Figure 1:**
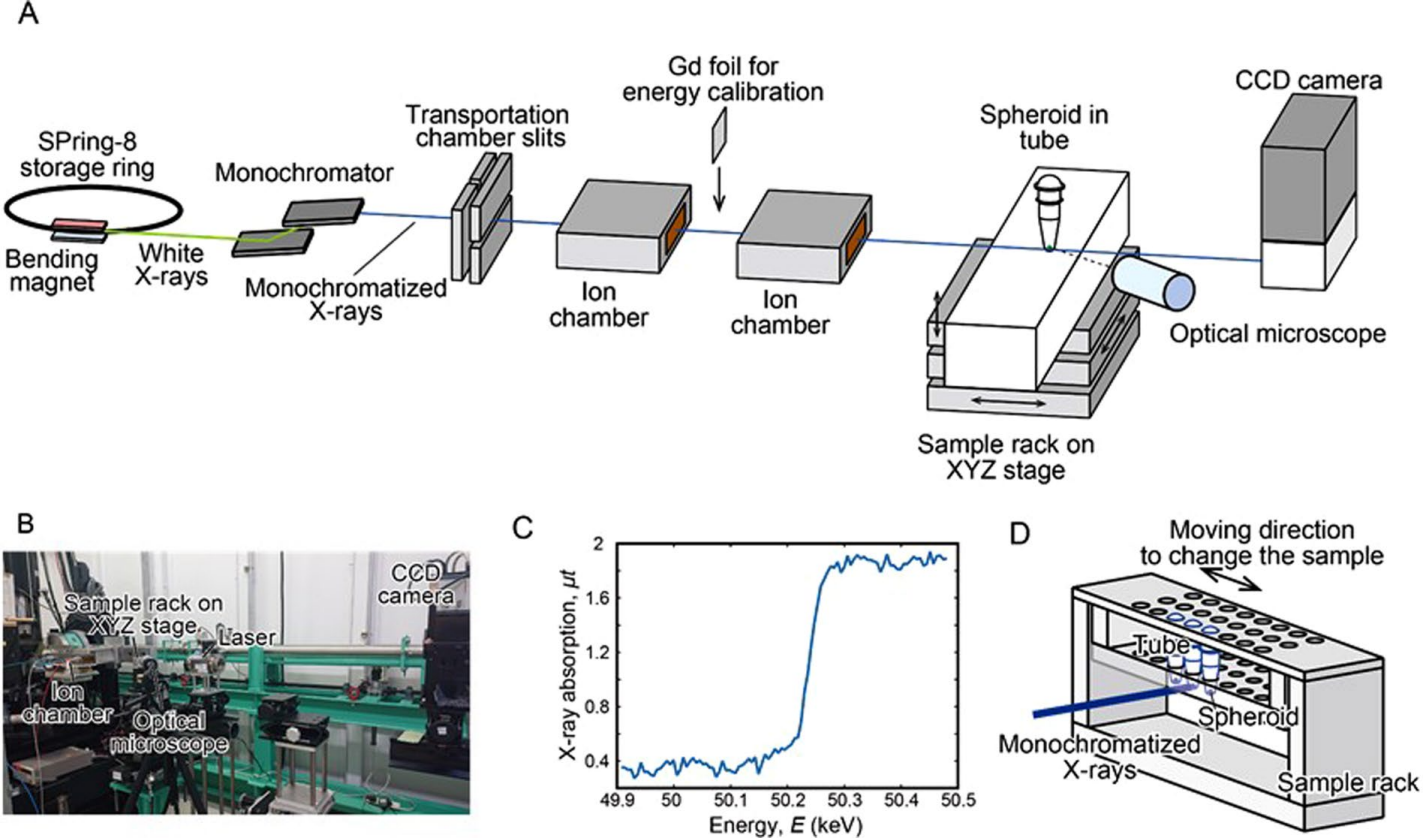
Experimental setup and X-ray K-edge absorption profile of gadolinium. Experimental setup for X-ray irradiation is shown in (**A**,**B** and **D**). (**C**) X-ray K-edge absorption profile was determined using gadolinium thin film.

Monochromatic X-ray experiments were carried out at a beamline BL14B1, SPring-8 synchrotron facility in Harima, Japan. The experimental setup is shown in Fig. 1. First, synchrotron radiation (SR) white X-rays from the bending magnet were led to a silicon double crystal monochromator to generate monoenergetic X-ray beam. The X-ray beam was shaped by using transport channel (TC) slits. The X-ray beam size was 1.4 mm × 1.4 mm at the sample position. This is enough to cover the tumor spheroid that has the dimension of 0.4 mm × 0.4 mm. The X-ray beam intensities were monitored by ion chambers during the experiment. The SPring-8 storage ring was operated in top-up-mode in which X-ray intensity fluctuations in time were negligible. The transmitted X-rays were monitored with CCD camera (Fig. 1A,B) to adjust the sample position. The spheroid is placed at the bottom of a tube that is placed in a sample rack (Fig. 1D). In a Monte Carlo simulation study to predict the effect of nanomaterials on the absorbed dose during an X-ray exposure^26^, gadolinium was shown to increase the absorbed dose.

To tune the energy of incident X-rays used in our experiment, we first measured X-ray K-edge absorption profile of gadolinium. A thin film of gadolinium 80 μm in thickness was irradiated by monochromatic X-rays of various energy levels and X-ray absorption was examined by the amount of X-rays that were transmitted through the foil. As shown in Fig. 1C, there was an abrupt increase in the amount of absorbed X-rays at 50.2 keV. This led us to use X-rays of 50.25 keV for the irradiation of tumor spheroids.

### Preparation of gadolinium loaded nanoparticles (Gd-MSN)

**Figure 2:**
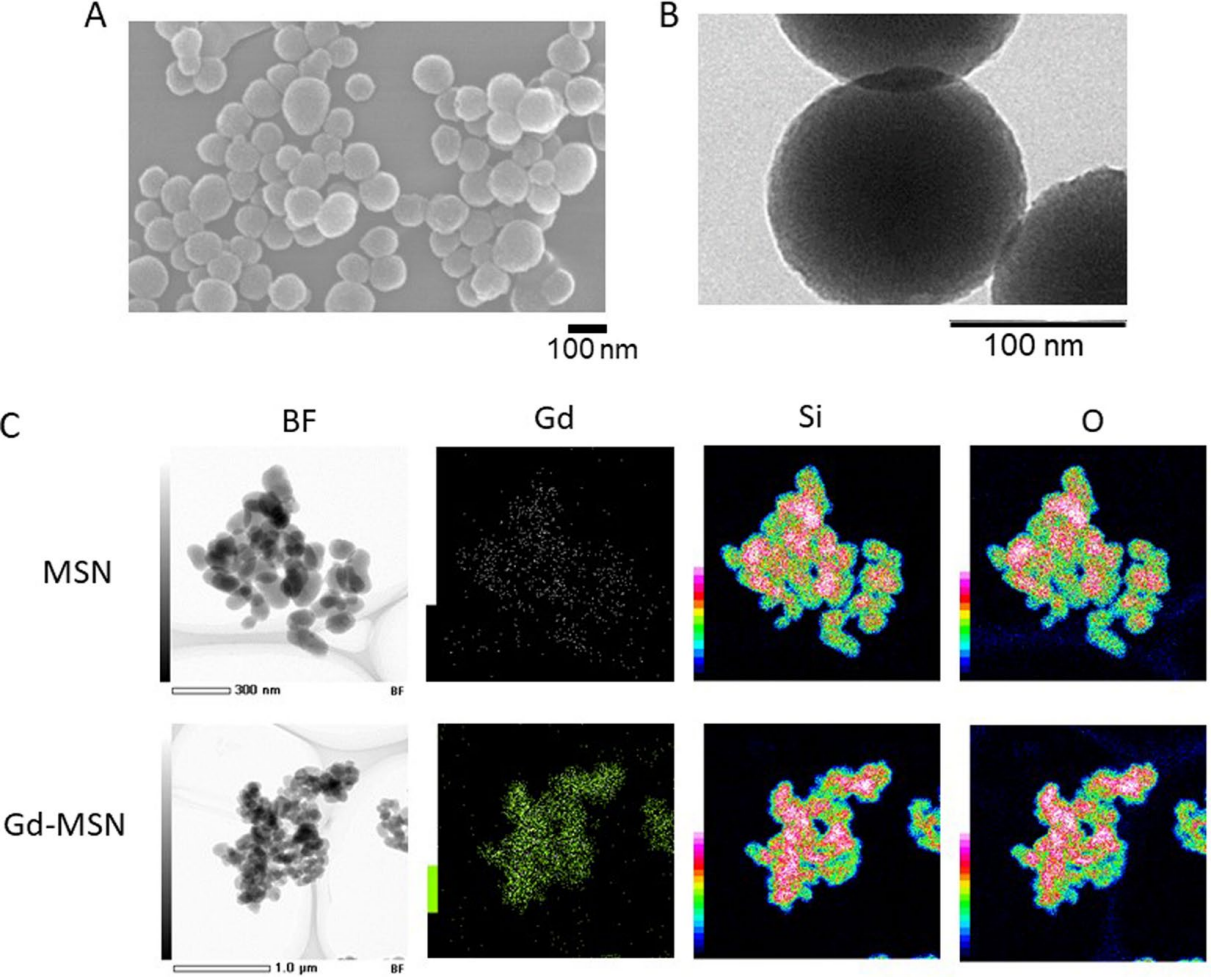
Characterization of nanoparticles. SEM (**A**) and TEM (**B**) results of NH_2_ and phosphonate surface modified MSN. (**C**) STEM-EDX images of Gd-MSN and MSN: STEM BF image and elemental mapping image by Gd, Si and O.

We prepared gadolinium loaded MSN by first synthesizing mesoporous silica nanoparticles (MSN) by the sol-gel method using CTAB as a templating agent^4^. Rhodamine-B was added during the synthesis to prepare fluorescent nanoparticles. Two types of surface modifications were carried out. First, we modified with phosphonate to achieve good dispersion of the nanoparticles. Second, we surface modified so that amine groups are present on the surface to enable loading of gadopentetic acid onto MSN as described by Kuthala *et al*.^27^. Figure 2A shows SEM characterization of MSN demonstrating homogeneous preparation of nanoparticles. The average diameter of MSN was 139 nm as examined by TEM (Fig. 2B). FT-IR results (Supplementary Fig. 1A) show a typical MSN band at 1050 cm^−1^. TGA analysis was consistent with the successful surface modification (Supplementary Fig. 1B). Nitrogen adsorption/desorption results (Supplementary Fig. 1C) are consistent with mesoporous materials and the pore diameter is calculated to be 3.5 nm (Supplementary Fig. 1D). Zeta potential of the Gd-MSN was −32.87 mV (Supplementary Fig. 2).

Figure 2C shows the scanning transmission electron microscopy-energy dispersive X-ray (STEM-EDX) mapping of gadolinium loaded MSN (Gd-MSN) and MSN. This technique can identify elemental composition of materials and the distribution of the elements in the materials. As can be seen, the distribution of Gd signal shown by green is detected on Gd-MSN but not on MSN. In contrast, we detected signals from Si and O on both nanoparticles similarly. This is because Si and O are components of the Si-O-Si framework of the nanoparticles. Based on the intensity of the signal, we estimate that the extent of Gd on the nanoparticle is 1–2% relative to the Si. ICP-AES analysis showed that the amount of gadolinium is 0.08 mg per 1 mg of MSN. Gadolinium was stably associated with MSN even after low pH exposure or sonication (Supplementary Figs 3, 4).

### Gd-MSN are efficiently taken up into cancer cells and are localized close to the cell nucleus

**Figure 3:**
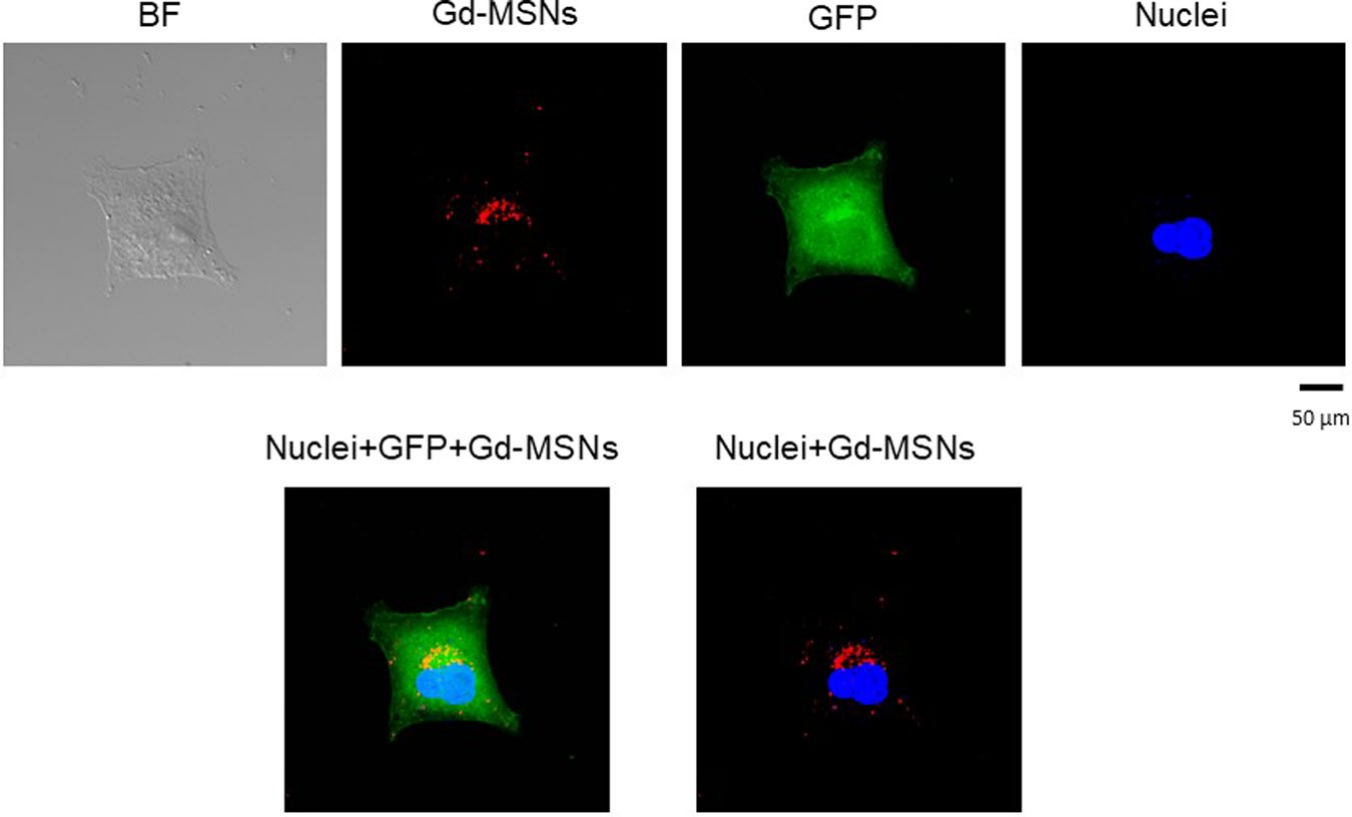
Uptake of Gd-MSN into human cancer cells. 20 ng of Gd-MSN (Rhodamine-B labeled) was incubated with ovarian cancer cells OVCAR8 that are engineered to express GFP for 24 hours. The cells were washed and examined by fluorescence microscopy. Nuclei were stained with Hoechst dye. BF is bright field.

We first examined uptake of Gd-MSN into cancer cells. Gd-MSN were added to the culture media of human ovarian cancer cells OVCAR8. After incubation for 1 day, the media were removed and the cells washed. The cells were examined by confocal microscopy. The nanoparticles were detected by red fluorescence and the cell nuclei were stained with Hoechst dye. Cancer cell cytoplasm was detected by green fluorescence, as these cells express GFP. As shown in Fig. 3, red fluorescence of the nanoparticles was detected just outside of the cell nucleus and was localized to one area of the nucleus. These results are consistent with lysosomal localization of MSN nanoparticles reported before^6,7^. We also examined potential cytotoxicity of Gd-MSN. Incubation with human embryonic kidney HEK293 cells or ovarian cancer OVCAR8 cells did not show any toxic effect up to 200 μg/ml (Supplementary Fig. 5).

### Uniform uptake of Gd-MSN into tumor spheroids

**Figure 4:**
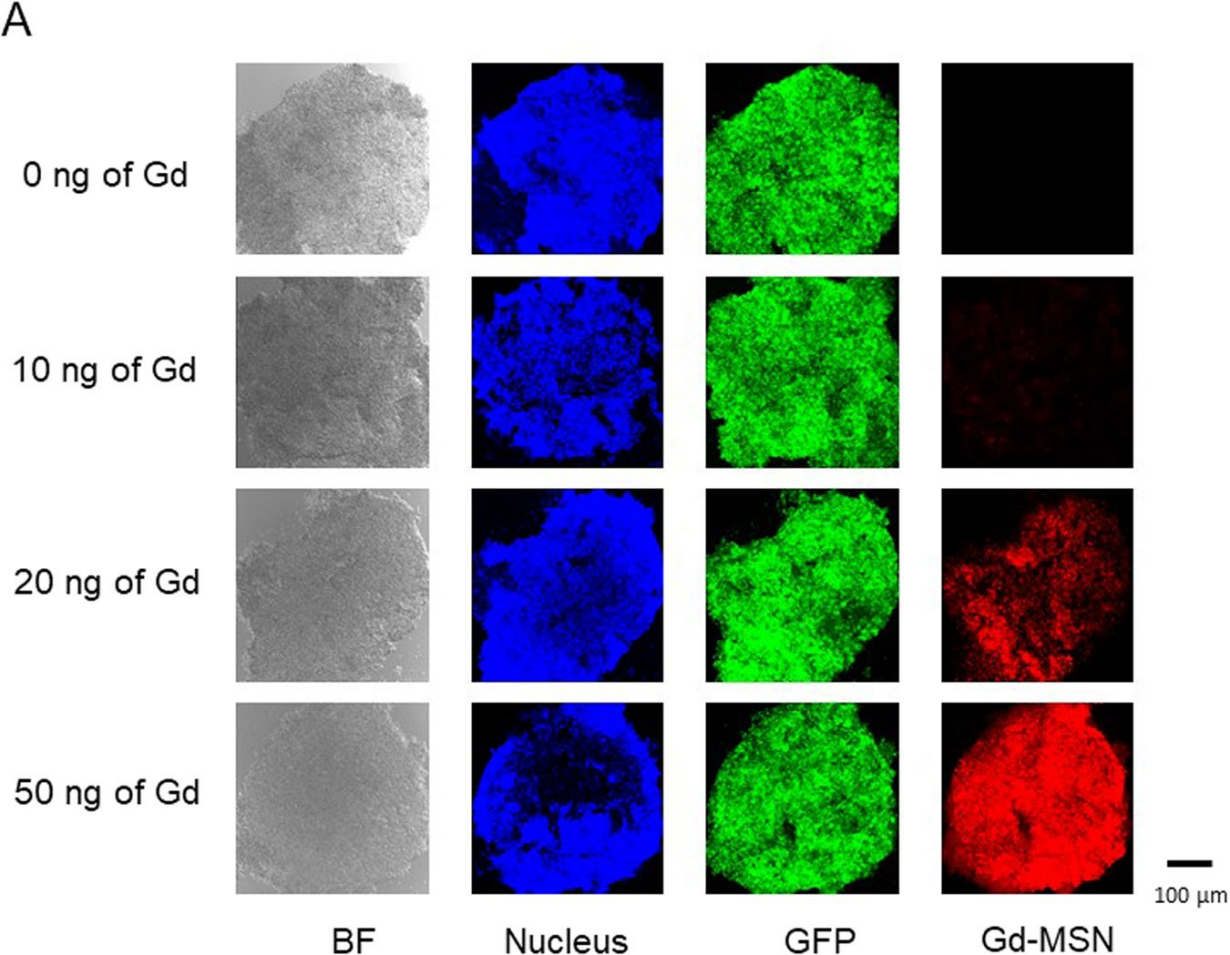
Uptake of Gd-MSN into tumor spheroids. Tumor spheroids were formed from OVCAR8 cells expressing GFP. Varying amounts of Rhodamine-B labeled Gd-MSN (0, 10, 20 or 50 ng of Gd) were added to the tumor spheroids and, after washing, fluorescence was measured. Cell nuclei were stained with Hoechst dye and thin sections were made by microtome and examined by confocal microscopy.

Tumor spheroids were prepared from ovarian cancer cells expressing GFP. This was accomplished by growing GFP-expressing ovarian cancer OVCAR-8 cells on a plate that has hydrophilic surface. Since the cells cannot stick to the plate surface, they are collected at the bottom of a well where they form three dimensional spheroids. When Gd-MSNs (Rhodamine-B labeled) were incubated with the tumor spheroids, we observed efficient uptake of nanoparticles as shown by red fluorescence on the spheroids. Cell nuclei were stained with Hoechst dye. The uptake of Gd-MSN was dependent on the amount of nanoparticles used to incubate with the spheroids as shown in Fig. 4.

**Figure 5:**
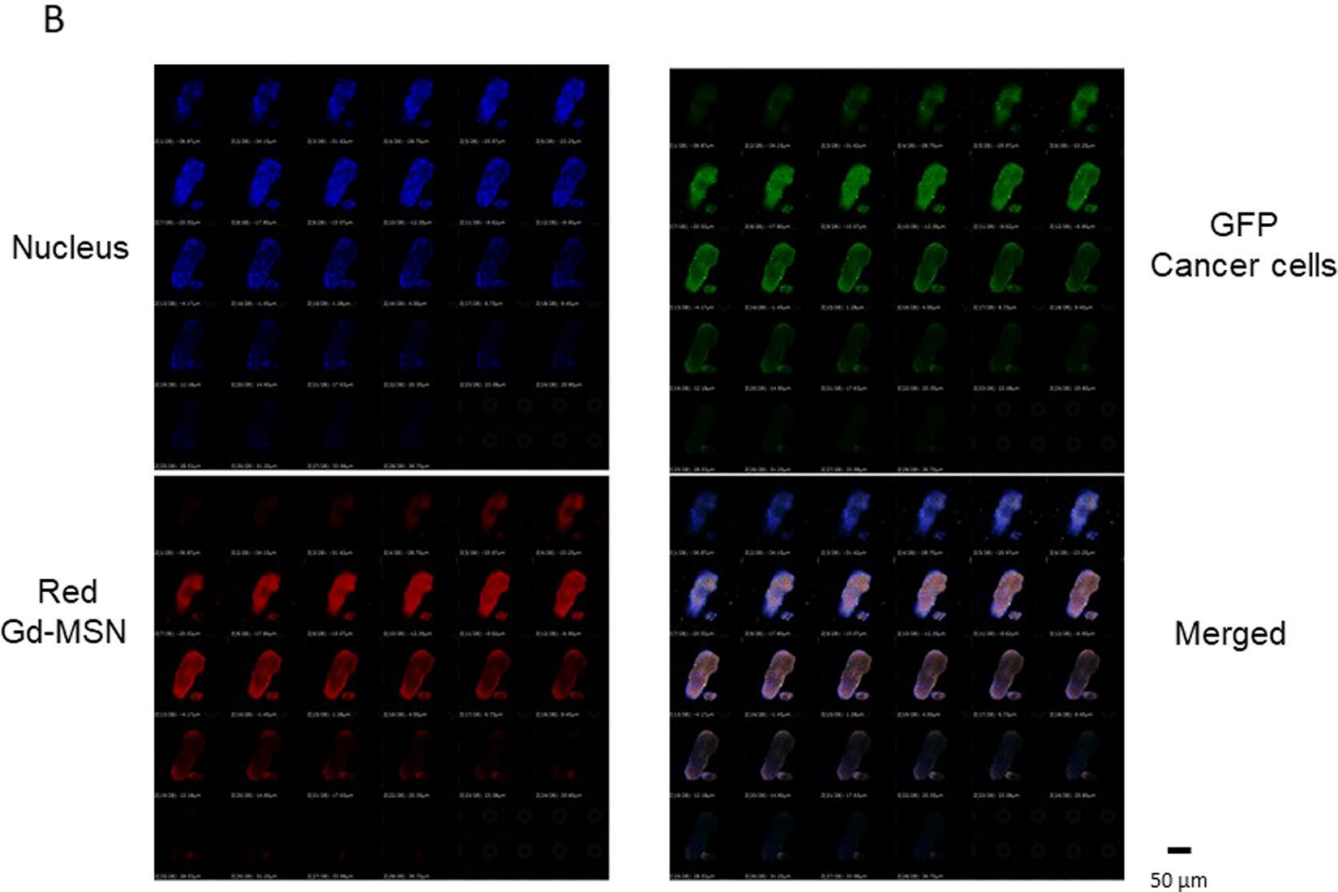
Analysis of Gd-MSN in tumor spheroids at various focal planes. Confocal microscopy was used to examine cancer cells (GFP) (green), Gd-MSN (red) and nuclei (blue) in tumor spheroids incubated with Gd-MSN at each focal plane. BF is bright field.

To confirm that Gd-MSN nanoparticles were taken up into cancer cells within the tumor spheroids, we carried out confocal microscopy (Fig. 5). Note that the cancer cells in the tumor spheroids are green as they express GFP. Cell nuclei were stained with Hoechst dye. As can be seen, red fluorescence of the nanoparticles overlapped well with the green fluorescence, suggesting that the nanoparticles are generally localized in the cancer cells within the tumor spheroids. The overlap of red and green fluorescence was observed in all focal planes. Thus, the nanoparticles are uniformly distributed in the tumor spheroids.

### Exposure to monochromatic X-rays: Tumor spheroid destruction is dependent on the irradiation time and the amount of gadolinium

**Figure 6:**
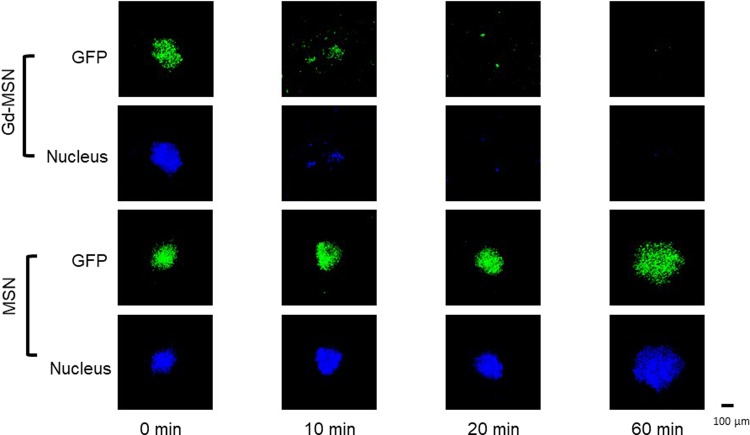
Effect of X-ray irradiation time on the destruction of tumor spheroids. OVCAR8 tumor spheroids incubated with Gd-MSN were irradiated with 50.25 keV X-rays for the indicated periods and destruction of tumor spheroids was examined. Spheroid destruction was followed by GFP fluorescence as well as by nuclear staining.

Exposure of tumor spheroids incubated with Gd-MSN to monochromatic X-rays was carried out at SPring-8 as described above. X-rays are guided to a station where the samples are placed. The stage is set up in a way that the irradiation position moves automatically to the next sample once the irradiation is completed (Fig. 1D). The photon flux at the sample position was calculated to be 3.11 × 10^8^ (photons/sec) using SPECTRA code^28^. After the irradiation, the tumor spheroids were incubated in a CO_2_ incubator and kept at 37 °C for three days and the spheroids were examined by visible and fluorescent microscopy (Fig. 6). The upper two columns show the experiments with tumor spheroids incubated with Gd-MSN while the lower two columns show the results with tumor spheroids incubated with empty MSN. Tumor spheroids were seen with green fluorescence of GFP expressing cancer cells. In addition, we stained cell nuclei with Hoechst dye. As can be seen, tumor spheroids incubated with Gd-MSN were broken up into pieces after 10 min exposure and no spheroids were observed after 60 min exposure. In contrast, tumor spheroids incubated with empty MSN appear intact even after 60 min irradiation. Note that the tumor spheroid destruction does not occur immediately but takes incubation for more than two days during which time cellular effect of irradiation becomes manifested (Supplementary Fig. 6).

**Figure 7:**
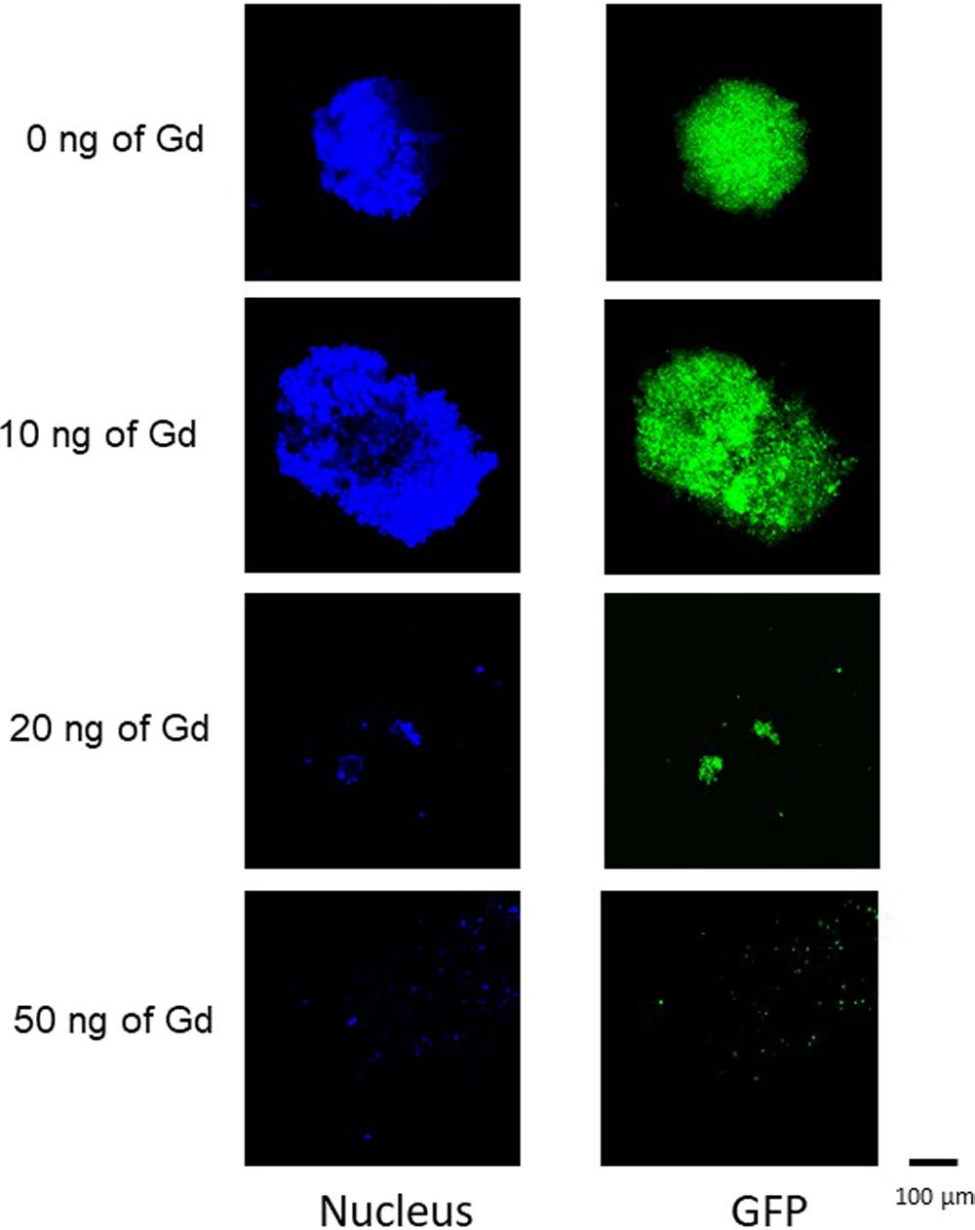
Effect of the amount of Gd-MSN incubated with tumor spheroids on the X-ray induced tumor spheroid destruction. OVCAR8 tumor spheroids incubated with 0, 10, 20, or 50 ng of Gd were irradiated with 50.25 keV X-rays and spheroid destruction was followed. GFP and nuclear staining were used to follow the destruction.

We also examined whether the tumor spheroid destruction is dependent on the amount of Gd-MSN used. Tumor spheroids were incubated with varying amounts of Gd-MSN, washed to remove nanoparticles that were not taken up, and then exposed to the X-ray beam. As can be seen in Fig. 7, tumor spheroids incubated with 50 ng of Gd were completely disintegrated after X-ray irradiation, while some fragments of the spheroids incubated with 20 ng of Gd were observable after the X-ray irradiation. The spheroids incubated with 10 ng of Gd retained their structure after the irradiation. Note that the extent of destruction is inversely proportional to the Gd-MSN loading shown in Fig. 4.

### The destruction of tumor spheroids requires K-edge energy of gadolinium

**Figure 8:**
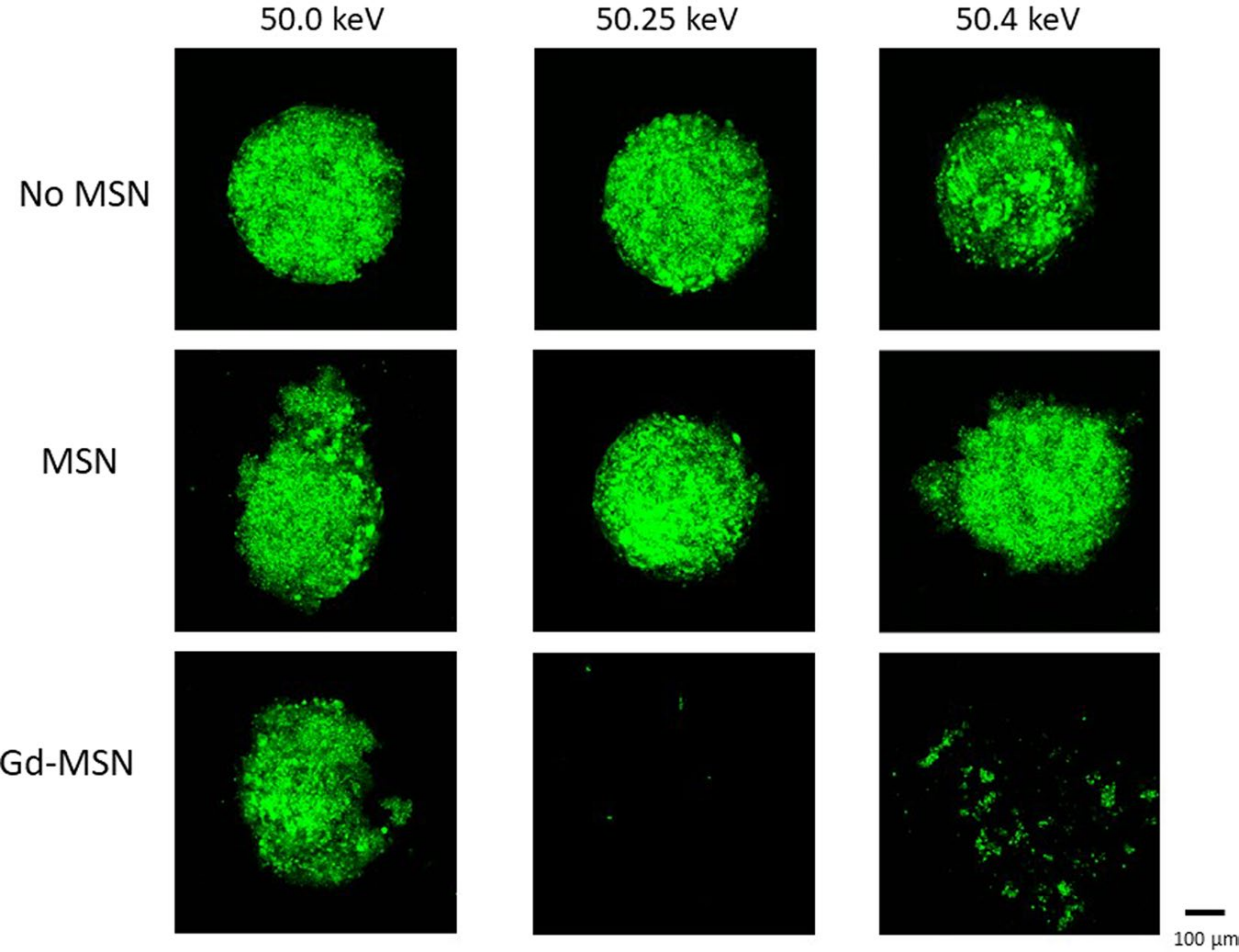
Energy dependence of tumor spheroid destruction. Tumor spheroids incubated with 50 ng of Gd-MSN were irradiated with X-rays with 50.0, 50.25 or 50.4 keV for 20 min. Tumor spheroid destruction was examined by GFP fluorescence.

A critical experiment is to examine X-ray energy dependence of the spheroid destruction. To examine this point, we opted to select X-ray beams with varying energy levels within a relative narrow range. Thus, the spheroids were irradiated with X-rays of 50.0, 50.25 or 50.4 keV. The results shown in Fig. 8 were quite striking, as almost no spheroid destruction was observed with X-rays of 50.0 keV, while the X-rays with 50.25 keV completely destructed the tumor spheroid. In the case of X-rays with 50.4 keV, the destruction was also observed. However, the extent of destruction was slightly less than that observed with the 50.25 keV X-rays, as some remaining tumor spheroids were detected.

## Discussion

It has been established that the exposure of high Z material to monochromatic X-rays that are designed to harbor K-shell edge energy results in the release of Auger electrons. These low energy electrons with limited flight path could damage DNA or other cellular components. However, demonstration of the cellular effect has not been easy to obtain. In our work, we used nanoparticles to facilitate uptake of high Z materials to cancer cells and to deposit them close to the cell nucleus. Using tumor spheroids loaded with gadolinium containing nanoparticles, we have demonstrated near complete destruction of the tumor spheroids when they were exposed to monochromatic X-rays of 50.25 KeV. This effect was exposure time dependent and was also dependent on the amount of gadolinium loaded to spheroids. Of critical importance is our observation that the spheroid destruction occurs when exposed to X-rays of 50.25 keV, while little destruction occurs when exposed to X-ray of 50.0 keV. This dramatic difference between the effect of 50.25 KeV and 50.0 KeV X-rays is consistent with the idea that the Auger electrons are exerting cellular effect.

When X-rays of 50.4 keV were used, tumor spheroid destruction also occurred, but we noted that the destruction efficiency was slightly less compared to what happens when expose to 50.25 keV X-rays (Fig. 8). From the absorption experiment, similar level of energy is likely to be absorbed. Therefore, the difference may be due to what happens after the energy absorption. The energy absorbed by high Z material will result in releasing electrons from the inner shell which then ignites electron release cascade. Perhaps this electron release process is efficient with 50.25 keV absorption but less at 50.4 keV X-ray absorption. In addition, energy of electrons released may be different between the two situations. It is also interesting that tumor spheroids were broken into pieces after the irradiation, which may suggest that the treatment has some effect on cell adhesion. Further work is needed to investigate these points.

Remarkable destruction of tumor spheroids upon X-ray exposure is likely due to the use of mesoporous silica nanoparticles to deliver gadolinium to cancer cells in tumor spheroids. We have shown that MSN are efficiently taken up into human cancer cells by endocytosis, an observation consistent with our previous resuls^6,7^. They are taken up into endosomes and end up in lysosomes that are located just outside of the cell nucleus. We also note that gadolinium loaded MSN are localized to one region just outside the cell nucleus. In addition to the proximity to DNA, this location has a number of organelles with vital cellular functions. This may have contributed to the effect we observed. Gd-MSN was uniformly distributed all over the spheroid, as demonstrated by the good overlap of green fluorescent cancer cells and red fluorescent nanoparticles, suggesting excellent penetration of the nanoparticles to tumor spheroids.

Conventional radiation therapy uses polychromatic X-ray beams that consist of a mixture of X-rays with different wave lengths. In contrast, use of synchrotron generated monochromatic X-ray beam has a number of advantages including the ability to design energy levels that match the target high Z material. While our results at this point are still limited to tumor spheroids, the use of nanoparticles opens up a possibility that a new type of radiation therapy can be developed. In a separate investigation, we have previously shown that MSNs have excellent tumor targeting capability in animal experiments; intravenous injection of MSN into the tail vein of xenograft mouse results in a predominant accumulation of MSN to the tumor^8^. Thus, we could envision a scenario where gadolinium is localized to the tumor first by the use of nanoparticles and then exposed to monochromatic X-ray beam to carry out tumor destruction. With respect to the X-ray source, one might be able to envision a compact synchrotron source as described by Eggl *et al*.^29^ in the future.

## Methods

### Synchrotron X-ray generation

Synchrotron radiation X-rays were generated from a bending magnet source. Synchrotron radiation white X-rays were monochromatized by a double-crystal fixed-exit monochromator with silicon 311 crystals. The SPring-8 storage ring was operated in top-up mode with a constant storage ring current of 100 mA. Monochromatized X-rays were shaped by a horizontal and a vertical slit. The incident beam size was 1.4 mm in height and 1.4 mm in width at the sample position. Two ion chambers were placed on the optical axis to monitor the incident beam intensity. Energy calibration of synchrotron radiation X-rays was done by measuring X-ray K-edge absorption profile of gadolinium foil, which was placed between the ion chambers. X-ray absorption *μt* was calculated by dividing *I* by *I*_0_, where *μ*, *t*, *I*, and *I*_0_ were linear absorption coefficient of gadolinium, thickness of gadolinium, the transmitted X-ray intensity, and the incident X-ray intensity, respectively.

### X-ray irradiation

Tubes containing spheroids were placed in a sample rack specially designed for the present experiment. The rack was located on a XYZ stage, which enables us to move samples on the optical axis for irradiation without entering the experimental hatch; series of irradiations can be done automatically. The sample position was checked before irradiation by using optical microscope and laser. It was also monitored by an X-ray CCD camera during irradiations. It is worth mentioning here that the energies of X-rays were so high that we could not observe the absorption contrast of the spheroids nor tubes by the CCD camera. Refraction-enhanced X-ray images of the tubes were obtained to monitor the sample positions.

### Synthesis of Gd-loaded MSN

Preparation of gadolinium-loaded MSN was carried out according to reported literature^27^ with slight modification. A mixture of 250 mg CTAB [Cetyltrimethylammonium bromide (CTAB, 98%), 3-(trihydroxysilyl)propyl methylphosphonate monosodium aqueous solution (50%), Sigma-Aldrich], 219 μL of 8 M NaOH solution and 120 mL of water was vigorously stirred at 80 °C. 2.5 mg of RITC [Rhodamine B isothiocyanate (RITC), Sigma-Aldrich] was dissolved in 5 mL ethanol and mixed with 6 μL of APTS [3-aminopropyltriethoxysilane (APTS, 99%), Wako] by stirring for 30 min at room temperature. After that, 1.2 mL of TEOS [Tetraethyl orthosilicate (TEOS, 95%), Wako] and 250 μL of APPS were mixed with the RITC-APTS solution and then added dropwise the CTAB solution. 315 μL of 3-(trihydroxysilyl)propyl methylphosphonate monosodium aqueous solution was added in the solution after 15 min. The solid material was collected by centrifugation and washed three times with ethanol. To remove CTAB, the as-synthesized solids were refluxed in a solution of concentrated HCl (2.3 mL) and ethanol (60 mL). The MSN-NH_2_ products were centrifuged, washed two times with ethanol and dried overnight. MSN-NH_2_ (10 mg) was ultrasonically dispersed in 0.1 M gadopentetic acid (Gd(III)DTPA) solution [Gadopentetic acid (Gd(III)DTPA, 97%, TRC) for 15 min. The solution was allowed to stir for 24 h. The Gd-MSN products were collected by centrifugation, respectively washed with water and ethanol to remove unreacted Gd(III)DTPA, and dried overnight.

Scanning Electron Microscopy (SEM) images were performed on a JEOL JSM-75FCT. Transmission Electron Microscopy (TEM) images were performed on a JEOL JEM-2100F. The scanning transmission electron microscopy-energy dispersive X-ray (STEM-EDX) analysis was carried out with a JEOL JEM-2200FS + JED2300T system operated at 200 kV. The quantitative measurement of the elements in the materials was performed by the Cliff-Lorimer ratio method to obtain the relative concentrations from the integrated EDX intensities. Zeta-potential measurements were carried out on ELS Z Otsuka Electronics. Fourier transform infrared (FT-IR) spectra were measured on a Bruker E400 FT-IR spectrometer using potassium bromide pellets. Thermal gravitational analysis (TGA) was recorded using a TA Instruments Q-500 thermal gravitational analyzer under airflow with a temperature ramp of 5 °C min^−1^. Low-pressure N_2_ adsorption measurements at 77 K were carried out on a Quantachrome Autosorb iQ volumetric gas adsorption analyzer. Helium was used as estimation of dead space. Ultrahigh-purity-grade N2, and He (99.999% purity) were used throughout adsorption experiments. ICP-AES (ICPE-9000 Shimadzu) was used to quantitate the amount of gadolinium loaded onto MSN.

### Tumor spheroids and incubation with Gd-loaded MSN

OVCAR8 (Human ovarian cancer) cells were grown on the 100 mm culture dish in RPMI1640 medium supplemented with 10% heat-inactivated FBS and 1% penicillin/streptomycin. 1.0 × 10^4^ of OVCAR8 cells were inoculated on PrimeSurface96U culture plate (MS-9096U, Sumitomo Bakelite Co., LTD., Japan) for spheroid formation. OVCAR8 cells were cultured for 7 days at 37 °C in a humidified CO_2_. The tumor spheroid diameter achieved was around 100–200 µm. Rhodamine B-labeled nanoparticles were added to tumor spheroids for 24 h at 37 °C in a humidified CO_2_. Each spheroid was collected into an eppendorf tube and centrifuged at 1500 rpm for 5 min. The supernatant was removed, and spheroids were washed with ice-cold PBS and were centrifuged at 1500 rpm for 5 min and fixed overnight with 4% paraformaldehyde at 4 °C. Spheroids were washed with ice-cold PBS and were treated with 99.8% methanol for 30 min at −80 °C. After washing spheroids, they were stained with Hoechst 33258 solution for 30 min in dark for confocal microscopy. For the analysis after irradiation, spheroids were incubated for 3 days and then processed for confocal microscopy.

## Supplementary information

Supplementary information

## Data Availability

Materials and data obtained in this study are deposited to the publisher site and are available upon request.
