# Supplementary material for: Destruction of tumor mass by gadolinium-loaded nanoparticles irradiated with monochromatic X-rays: Implications for the Auger therapy
Source: Sci Rep. 2019 Sep 30;9:13275. doi: 10.1038/s41598-019-49978-1 (PMC6768997; doi:10.1038/s41598-019-49978-1)
Supplement: Supplementary file 1 — Supplementary information [file 41598_2019_49978_MOESM1_ESM.pdf]

Supplementary Information

Destruction of tumor mass by gadolinium-loaded nanoparticles irradiated with monochromatic X-rays: Implications for the Auger therapy

Kotaro Matsumoto, Hiroyuki Saitoh, Tan Le Hoang Doan, Ayumi Shiro,
Keigo Nakai, Aoi Komatsu, Masahiko Tsujimoto, Ryo Yasuda,
Tetsuya Kawachi, Toshiki Tajima
and Fuyuhiko Tamanoi

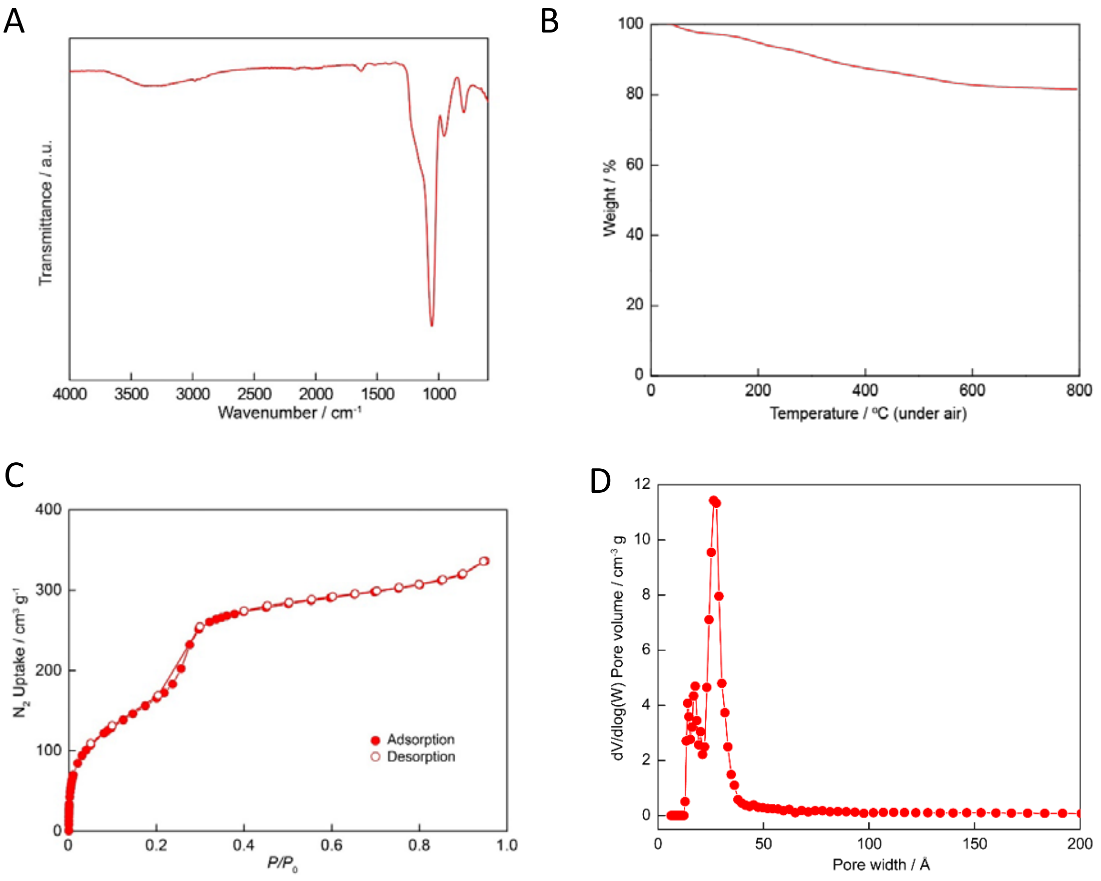

Supplementary Figure 1: Characterization of MSN before gadolinium loading. A: FT-IR analysis. B: TGA (thermogravitational analysis). C: Nitrogen adsorption/desorption analysis. D: Pore size distribution.

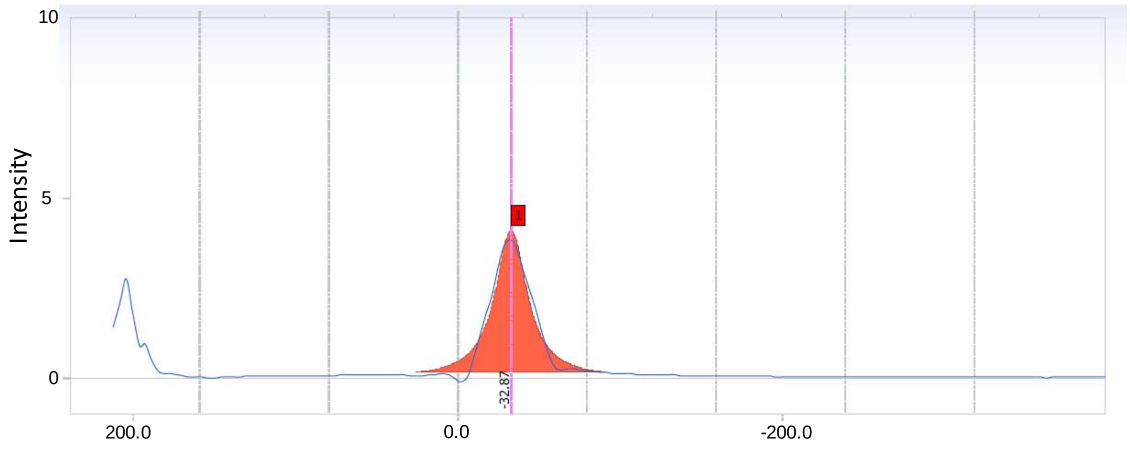

Supplementary Figure 2: Zeta potential of Gd-MSN.

Treatment	Amount of Gd on MSN (ppm)
Control	1.04
pH 5.5	1.03
pH 6.0	0.95
pH 6.5	0.96

Supplementary Figure 3: Gadolinium remains bound to MSN after incubation in low pH solutions. Incubation was for two hours. The amount of gadolinium was examined by ICP-AES.

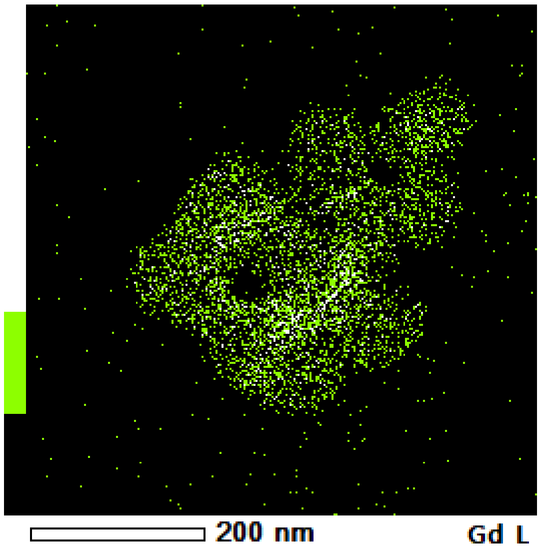

Supplementary Figure 4: STEM-EDX analysis of Gd-MSN after sonication for 30 min.

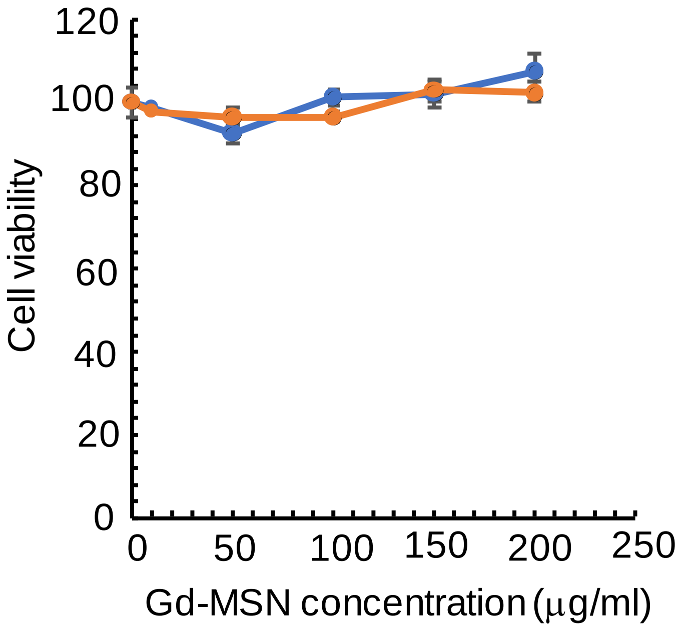

Supplementary Figure 5: Lack of toxic effect of Gd-MSN on HEK293 (orange) and OVCAR8 (blue) cells.

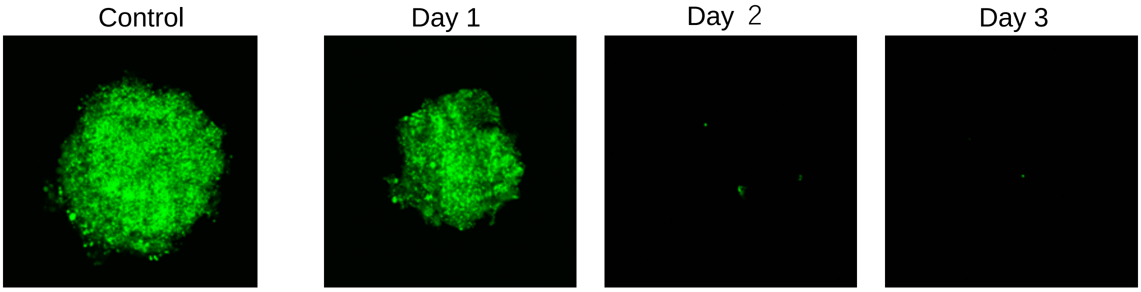

Supplementary Figure 6: Time course of spheroid destruction after irradiation.
